# Tannic acid-modified silver nanoparticles enhance the anti-*Acanthamoeba* activity of three multipurpose contact lens solutions without increasing their cytotoxicity

**DOI:** 10.1186/s13071-020-04453-z

**Published:** 2020-12-22

**Authors:** Edyta B. Hendiger, Marcin Padzik, Agnieszka Żochowska, Wanda Baltaza, Gabriela Olędzka, Diana Zyskowska, Julita Bluszcz, Sylwia Jarzynka, Lidia Chomicz, Marta Grodzik, Jacek Hendiger, José E. Piñero, Jarosław Grobelny, Katarzyna Ranoszek-Soliwoda, Jacob Lorenzo-Morales

**Affiliations:** 1 Laboratory of Parasitology, Department of Medical Biology, Medical University of Warsaw, 14/16 Litewska Street, 00-575 Warsaw, Poland; 2 Instituto Universitario de Enfermedades Tropicales y Salud Pública de Canarias and Departamento de Obstetricia, Ginecología, Pediatría, Medicina Preventiva y Salud Pública, Toxicología, Medicina Legal y Forense y Parasitología, Universidad de La Laguna, Avenida Astrofísico Francisco Sánchez S/N, 38203 Tenerife, Spain; 3 Department of Nanobiotechnology and Experimental Ecology, Institute of Biology, Warsaw, University of Life Sciences, 8 Ciszewskiego Street, 02-787 Warsaw, Poland; 4 Faculty of Building Services, Hydro and Environmental Engineering, Warsaw University of Technology, 20 Nowowiejska Street, 00-653 Warsaw, Poland; 5 Department of Materials Technology and Chemistry, Faculty of Chemistry, University of Lodz, 163 Pomorska Street, 90-236 Lodz, Poland

**Keywords:** *Acanthamoeba* keratitis, Contact lens solutions, Silver nanoparticles, Tannic acid

## Abstract

**Background:**

Free-living amoebae of the genus *Acanthamoeba* are cosmopolitan, widely distributed protozoans that cause a severe, vision-threatening corneal infection known as *Acanthamoeba* keratitis (AK). The majority of the increasing number of AK cases are associated with contact lens use. Appropriate eye hygiene and effective contact lens disinfection are crucial in the prevention of AK because of the lack of effective therapies against it. Currently available multipurpose contact lens disinfection systems are not fully effective against *Acanthamoeba* trophozoites and cysts. There is an urgent need to increase the disinfecting activity of these systems to prevent AK infections. Synthesized nanoparticles (NPs) have been recently studied and proposed as a new generation of anti-microbial agents. It is also known that some plant metabolites, including tannins, have anti-parasitic activity. The aim of this study was to evaluate the anti-amoebic activity and cytotoxicity of tannic acid-modified silver NPs (AgTANPs) conjugated with selected multipurpose contact lens solutions.

**Methods:**

The anti-amoebic activities of pure contact lens care solutions, and NPs conjugated with contact lens care solutions, were examined in vitro by a colorimetric assay based on the oxido-reduction of alamarBlue. The cytotoxicity assays were performed using a fibroblast HS-5 (ATCC CRL-11882) cell line. The results were statistically analysed by ANOVA and Student-Newman-Keuls test using *P* < 0.05 as the level of statistical significance.

**Results:**

We show that the NPs enhance the anti-*Acanthamoeba* activities of the tested contact lens solutions without increasing their cytotoxicity profiles. The activities are enhanced within the minimal disinfection time recommended by the manufacturers.

**Conclusions:**

The conjugation of the selected contact lens solutions with AgTANPs might be a novel and promising approach for the prevention of AK infections among contact lens users.
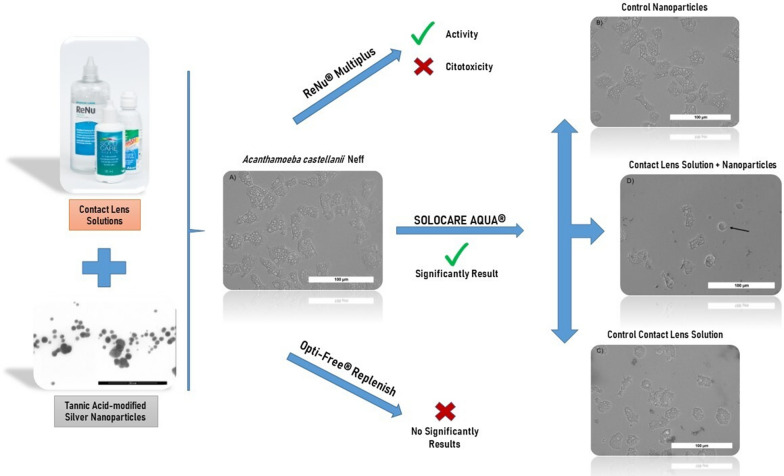

## Background

Amoebae of the genus *Acanthamoeba* are free-living, abundant and cosmopolitan protozoans that show various degrees of pathogenicity to humans. They are ubiquitous in both natural and manmade environments. As facultative human parasites, when transmitted from the environment to the eye surface, they may cause a progressive, sight-threatening corneal infection known as *Acanthamoeba* keratitis (AK) [1–5]. Improper use and disinfection of contact lenses, corneal damage, and exposure of the eyes to water polluted with *Acanthamoeba* are the primary risk factors of AK. The lack of specific symptoms in the early stages of the infection, and co-infections with other microorganisms, cause serious diagnostic difficulties and a delay in treatment. The number of AK infections has been increasing worldwide. Current therapeutic approaches are limited to the prolonged application of diamidines and biguanides. However, these treatments are not specific and are very toxic to the eye [6–9]. Amoebic trophozoites may attach to the surface of both contact lenses and contact lens storage cases. Multipurpose contact lens disinfection systems are not effective against *Acanthamoeba* and their anti-amoebic activity needs improvement [10–12]. In summary, prevention, including proper eye hygiene and effective contact lens disinfection, seems to be the best approach to limiting the incidence of AK.

In recent years, the fast development of nanotechnology has been observed. Synthetised nanoparticles (NPs) are currently proposed as a new generation of anti-bacterial, anti-viral and anti-fungal agents [13, 14]. Moreover, NP activity against different protozoans such as *Giardia intestinalis*, *Entamoeba histolytica*, *Cryptosporidium parvum* and *Leishmania* spp. has been already confirmed [15–17]. Plant metabolites, including tannins, present anti-microbial activity [18, 19]. They are capable of forming insoluble complexes with nucleic acids, carbohydrates, proteins and chelating metal ions. Tannic acid (penta-*m*-digalloyl glucose) is the simplest polyphenolic, hydrolysable plant metabolite with confirmed anti-bacterial, anti-cancer and anti-oxidant activity [20–23]. In our previous studies we demonstrated that tannic acid-modified silver NPs (AgTANPs) were well absorbed and showed anti-amoebic activity against *Acanthamoeba* strains belonging to the T4 genotype [24]. Other authors have confirmed that NPs enhance the anti-amoebic effect of biguanides such as chlorhexidine digluconate and other therapeutic compounds [25–27]. The aim of this study was to evaluate the activity and cytotoxicity of AgTANPs conjugated with selected multipurpose contact lens solutions against the trophozoite stage of a strain of *Acanthamoeba castellanii* belonging to the T4 genotype.

## Methods

### Cultivation of the strain

*Acanthamoeba castellanii* Neff strain ATCC 30010 was cultured axenically in 25-cm^2^ culture tissue flasks, without shaking, at 27 °C in PYG medium [0.75% (w/v) proteose peptone, 0.75% (w/v) yeast extract and 1.5% (w/v) glucose] containing gentamicin (10 mg/ml), at the Department of Medical Biology, Medical University of Warsaw, Poland. The culture was sub-cultured twice a month and growth assessed using direct light microscopy and a Bürker chamber (haemocytometer).

### Nanoparticles

**Fig. 1:**
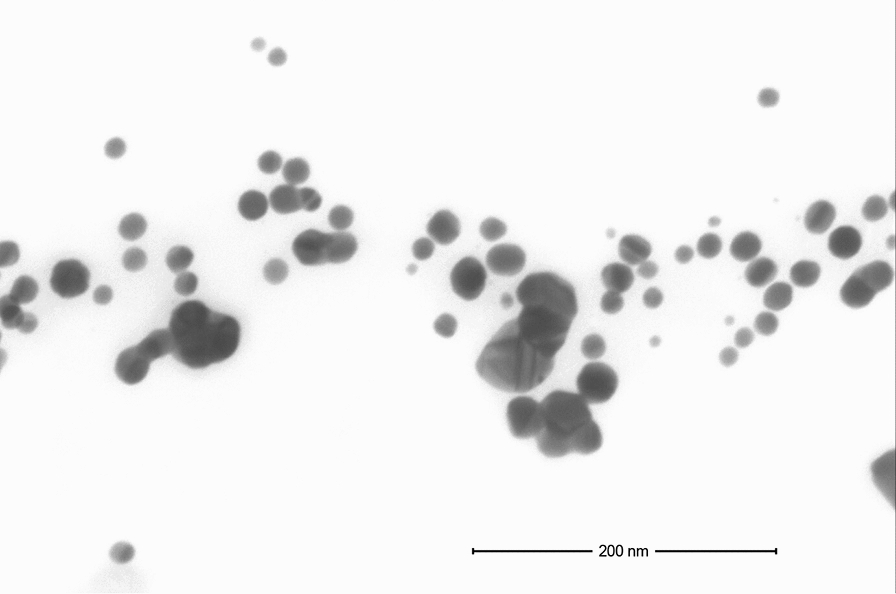
High-resolution scanning transmission electron microscopy image of the distribution and diameters of the tannic acid-modified silver nanoparticles (AgTANPs)

AgTANPs were synthesized by a chemical reduction method using silver nitrate (AgNO_3_; purity 99.999%; Sigma-Aldrich, St Louis, MO). AgTANPs were prepared by mixing a heated aqueous solution of AgNO_3_ (95.2 g, 0.017%) with an aqueous solution of a tannic acid (0.6 g, 5% C_76_H_52_O_46_; Sigma-Aldrich). The long-term stability of the colloidal dispersions of all tested NPs (ζ potential) was measured and confirmed by the electrophoretic light-scattering method with a Zetasizer Nano ZS (model ZEN3500; Malvern Instruments, Worcestershire, UK) [26, 28]. The size and shape of the AgTANPs were determined by high-resolution scanning transmission electron microscopy (HR-STEM) (Fig. 1). Measurements were taken with a scanning electron microscope (Nova NanoSEM 450; FEI) using transmission mode (STEM II) at an accelerating voltage of 30 kV. Samples for HR-STEM investigations were prepared as follows: a drop of colloid was deposited onto carbon-coated copper grids (300 mesh) and left for 2 h for solvent evaporation. The well-dispersed nanofluids were used as a stock solution and were appropriately diluted to various concentrations ranging between 0.25–2.5 p.p.m. and used in subsequent activity and cytotoxicity assays.

### Contact lens solutions

The multipurpose solutions used in this study represent the three most common types of solutions used for contact lens care in Poland, namely Solo Care Aqua (SCA), Opti-Free (O-F) and ReNu MultiPlus (ReNu). The tested contact lens care solutions and their ingredients are included in Table 1. All multipurpose solutions used in the study were purchased from authorized agents.

**Table 1:** Ingredients of the multipurpose contact lens solutions and minimum disinfection times recommended by the manufacturers

Manufacturer	Solution	Ingredients	Minimum disinfection time (h)
Bausch + Lomb	ReNu MultiPlus (ReNu)	Hydranate (hydroxyalkylphosphonate) 0.03%, boric acid, edetate disodium, poloxamine 1%, sodium borate, sodium chloride, preserved with Dymed (polyaminopropyl biguanide 0.0001%)	4
Alcon	Opti-Free (O-F) (RepleniSH)	TearGlyde (Tetronic 1304, nonanoyl ethylenediaminetriacetic acid), Polyquad (polyquaternium-1) 0.001%, Aldox (myristamidopropyl dimethylamine) 0.0005%	6
Menicon	Solo Care Aqua (SCA)	Polyhexanide 0.0001%, Hydrolock (dexpanthenol, sorbitol), sodium phosphate, tromethamine, poloxamer 407, disodium edetate	4

### Activity assays

Pure contact lens solutions, and NPs at concentrations of 0.25, 0.5, 1.25 and 2.5 p.p.m. conjugated with the contact lens care solutions, were examined in vitro and assessed for their anti-amoebic activity. To determine the anti-amoebic efficacy on trophozoites (log growth phase after 6 days following sub-culturing), the previously described colorimetric 96-well microtitre plate assay, based on the oxido-reduction of alamarBlue, was used [29]. Subsequently, the plates were analysed over a period of 6 h, 24 h, 48 h, 72 h and 96 h in a Synergy HTX Multimode Microplate Reader (BioTek) using the Gen5 software programme, a test wavelength of 570 nm and a reference wavelength of 630 nm in order to calculate the inhibition curves of the analysis. All experiments were performed three times, in triplicate. Amoebae growth and viability (trophozoite movement and presence of acanthopodia) in both control and tested assays were visualized by an Evos FLoid Cell Imaging System (ThermoFisher).

### Cytotoxicity

Briefly, the cytotoxicity assays were performed using a fibroblast HS-5 (ATCC CRL-11882) cell line as described in our previous study [24]. A commercial kit for the evaluation of drug-induced cytotoxic effects based on the measurement of lactate dehydrogenase (LDH) activity released to the media (Pierce LDH cytotoxicity assay kits 88953, 88954) was used as per protocol. The fibroblasts were incubated with each of the contact lens solutions separately and the contact lens solution plus NPs added at the same concentration as in the activity assays. To calculate the percent cytotoxicity, absorbance was measured at 490 nm and 680 nm.

### Statistical analysis

All experiments were performed three times in triplicate. SD and mean values were calculated for all activity and cytotoxicity data. The results were statistically analysed by ANOVA and Student-Newman–Keuls test using *P* < 0.05 as the level of statistical significance.

**Table 2:** Anti-amoebic activity of the pure contact lens solutions and tannic acid-modified silver nanoparticles (*AgTANPs*) conjugated with the contact lens solutions after 6–96 h of incubation (% inhibition)

	6 h	24 h	48 h	72 h	96 h
SCA	31.95 ± 1.70	23.15 ± 3.93	37.68 ± 1.16	51.65 ± 2.75	47.15 ± 3.25
SCA + 2.5 p.p.m. AgTANPs	61.18 ± 1.34	51.60 ± 8.50	59.79 ± 11.19	66.02 ± 5.42	61.9 ± 2.41
SCA + 1.25 p.p.m. AgTANPs	61.67 ± 4.63	42.79 ± 19.26	52.03 ± 11.57	60.42 ± 4.25	54.50 ± 0.21
SCA + 0.5 p.p.m. AgTANPs	54.91 ± 3.89	36.88 ± 20.08	47.33 ± 10.25	58.21 ± 3.07	72.77 ± 1.71
SCA + 0.25 p.p.m. AgTANPs	52.49 ± 6.35	35.45 ± 10.65	45.35 ± 6.56	55.95 ± 1.15	51.31 ± 1.13
O-F	No activity	No activity	No activity	35.74 ± 0.95	47.35 ± 2.75
O-F 2.5 p.p.m. AgTANPs	31.11 ± 3.09	21.83 ± 4.85	44.79 ± 4.92	59.58 ± 1.14	57.72 ± 0.55
O-F + 1.25 p.p.m. AgTANPs	No activity	4.50 ± 11.51	33.32 ± 4.38	52.78 ± 0.98	52.29 ± 0.24
O-F + 0.5 p.p.m. AgTANPs	No activity	No activity	16.63 ± 3.70	41.62 ± 3.08	41.15 ± 2.46
O-F + 0.25 p.p.m. AgTANPs	No activity	No activity	No activity	36.82 ± 1.69	36.74 ± 1.77
ReNu	No activity	No activity	24.23 ± 4.88	43.99 ± 2.10	46.76 ± 0.64
ReNu 2.5 p.p.m. AgTANPs	41.59 ± 2.18	36.63 ± 4.69	56.72 ± 3.58	67.45 ± 2.68	64.59 ± 2.98
ReNu + 1.25 p.p.m. AgTANPs	39.58 ± 2.66	31.17 ± 5.28	50.07 ± 3.57	62.77 ± 2.79	58.85 ± 3.63
ReNu + 0.5 p.p.m. AgTANPs	39.18 ± 0.87	22.60 ± 7.05	45.22 ± 5.33	61.90 ± 2.21	58.56 ± 3.39
ReNu + 0.25 p.p.m. AgTANPs	32.99 ± 4.42	13.47 ± 0.14	43.52 ± 4.20	62.12 ± 7.68	57.55 ± 0.11

*No activity* indicates a statistically insignificant result (*P* > 0.05). For other abbreviations, see Table 1

## Results

### Activity

Our initial results confirmed the insufficient anti-amoebic effect of the tested contact lens solutions against *Acanthamoeba* trophozoites. Anti-amoebic activity was revealed for SCA and reached 32% inhibition after 6h of incubation. ReNu and O-F did not show an anti-amoebic effect on the tested *Acanthamoeba* strain within the first 24 h of incubation. The detailed data are shown in Table 2.

**Fig. 2:**
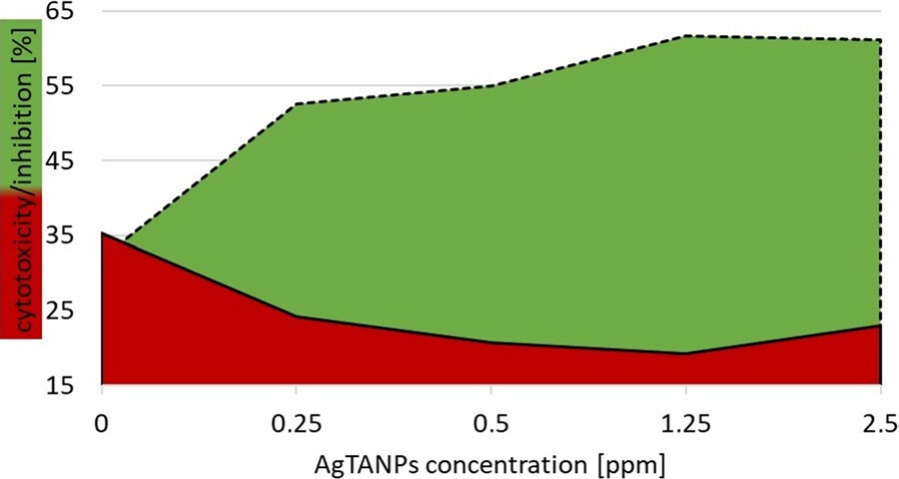
Anti-*Acanthamoeba* activity of AgTANPs conjugated with Solo Care Aqua (SCA) contact lens solution after 6 h of incubation in relation to cytotoxicity

**Fig. 3:**
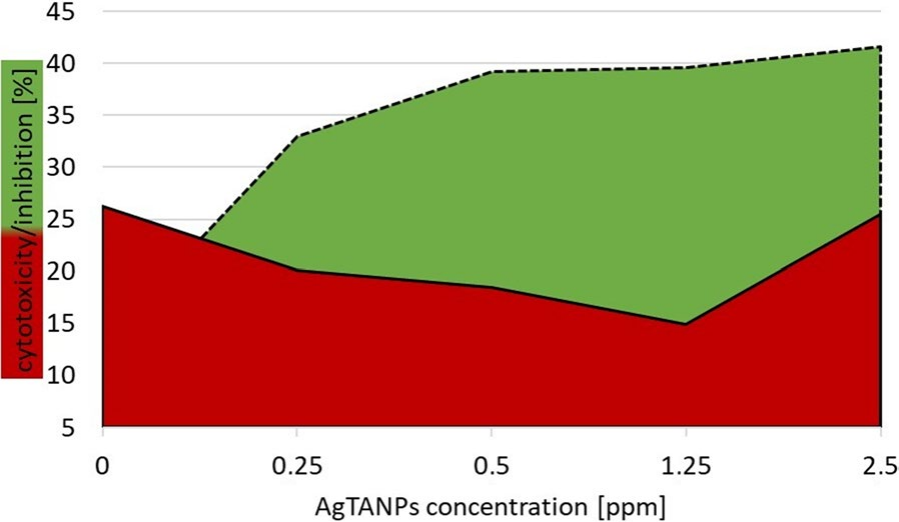
Anti-*Acanthamoeba* activity of AgTANPs conjugated with ReNu MultiPlus contact lens solution after 6 h of incubation in relation to cytotoxicity

**Fig. 4:**
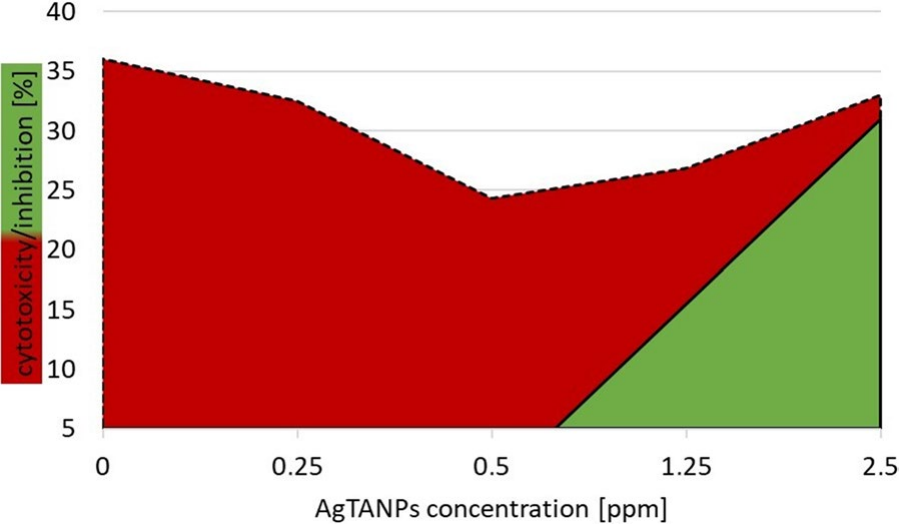
Anti-*Acanthamoeba* activity of AgTANPs conjugated with Opti-Free contact lens solution after 6 h of incubation in relation to cytotoxicity

AgTANPs significantly enhanced anti-*Acanthamoeba* activity of the tested contact lens solutions. Specifically, AgTANPs conjugated with SCA showed the most promising dose-dependent increase of amoebae inhibition after the minimal disinfection time recommended by the manufacturers (6 h) (Fig. 2). A similar anti-amoebic effect was achieved for AgTANPs conjugated with ReNu (Fig. 3). The enhanced anti-amoebic effect of both conjugates lasted up to 96 h of incubation. O-F conjugated with the NPs did not show any enhanced effect during the first 24 h of incubation (Fig. 4). The anti-amoebic effects were revealed just after 48 h of incubation. The detailed results are shown in Table 2.

**Fig. 5:**
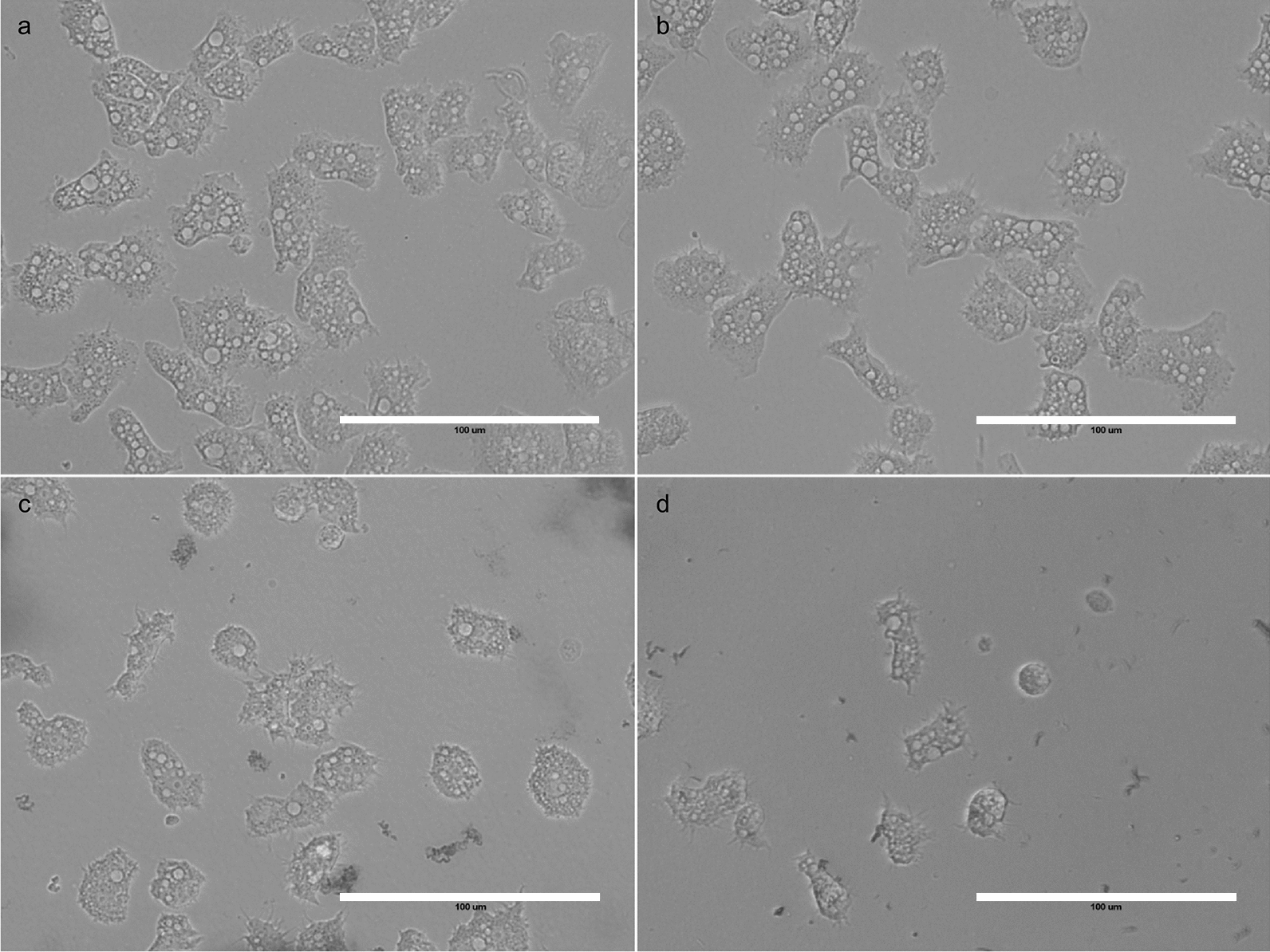
**a**–**d** *Acanthamoeba* trophozoites after 6 h of incubation. **a** Control culture in PYG medium. **b** Incubation with AgTANPs. **c** Incubation with SCA. **d** Incubation with AgTANPs conjugated with SCA. The* arrow* shows a rounded form. All images (× 40) represent the population of treated amoebae and were taken under a live cell imaging microscope (EVOS FLoid Cell Imaging Station). For abbreviations, see Figs. 1 and 2

Compared to the control culture (Fig. 5a), 6 h of incubation with AgTANPs did not influence the morphology or the viability of the amoebae at the level of microscopic observation used here. Incubation with SCA caused decreased mobility of the trophozoites. Observed acanthopodia were less extensive than in the control cultures. Fragments of the disrupted cells were visualized between viable trophozoites (Fig. 5a). After 6 h of incubation with AgTANPs conjugated with SCA, morphological degeneration of the trophozoites developed (Fig. 5d). The size of the cells and the number of visible acanthopodia were lower compared to the assays illustrated in Fig. 5a–c. There were more disrupted cell fragments visualized. Some trophozoites started developing into rounded forms.

### Cytotoxicity

The overall cytotoxicity measured for SCA and O-F was similar and reached 36%. The cytotoxicity of ReNu reached 26%. Cytotoxicity values for NPs conjugated with the contact lens solutions were not statistically significantly different from those of the pure contact lens solutions. The cytotoxicity results are listed in Table 3.

**Table 3:** Cytotoxicity of the contact lens solutions (*CLS*) and the CLS conjugated with the AgTANPs (%)

	CLS	CLS + 0.25 p.p.m. AgTANPs	CLS + 0.5 p.p.m. AgTANPs	CLS + 1.25 p.p.m. AgTANPs	CLS + 2.5 p.p.m. AgTANPs
SCA	35.3	24.2	20.7	19.2	23.0
O-F	36.0	32.5	24.3	26.9	33.0
ReNu	26.2	20.1	18.5	15	25.5

For other abbreviations, see Tables 1 and 2

## Discussion

In recent years cases of AK have been increasingly diagnosed worldwide. The available anti-amoebic therapies are not fully effective against AK, and often result in damaging cytotoxicity to the human eye. The main key predisposing factor for AK is contact lens use. Effective contact lens disinfection is the best approach to minimising the incidence of AK. In this study, we tested multipurpose contact lens disinfecting systems containing different active ingredients, but characterized by a similar mode of action, which result in cell membrane perturbation (Table 1). Our results confirmed a lack of amoebicidal activity for all the tested multipurpose contact lens solutions against the *Acanthamoeba* strain used here, in accordance with other publications revealing that the disinfecting capabilities of current contact lens solutions are insufficient [11, 12, 30–33].

Rapid developments in nanotechnology have significantly improved the anti-microbial potential of NPs, especially silver NPs (AgNPs) [14, 34, 35]. The specific mechanism of action of AgNPs is still not entirely understood; however, recent studies conducted on bacteria did shed more light on this process. We know that NPs cause damage to the cell membrane. Adhesion of NPs is based on the electrostatic attraction of the negatively charged cell membrane and positively or less negatively charged NPs. The interaction decreases the ζ potential and depolarizes the cell membrane. This process leads to the disruption of membrane permeability and an alteration of the respiratory functions of the cell, eventually leading to disruption of cell integrity [36]. After crossing the cell membrane, NPs can interact with DNA, RNA and proteins, altering both transcription and translation processes. The presence of NPs in the cell causes oxidative stress and disruption of enzymatic pathways due to the resultant free radicals. Altogether, NPs cause cytotoxic effects and finally lead to cell death. The cytotoxicity of AgNPs depends on their physicochemical properties such as size and density. Typically, smaller NPs have a relatively increased stability and enhanced anti-microbial activity. Similarly, higher concentrations of NPs show increased anti-microbial activity. However, this property is strictly correlated to the tested microbial species and the type of NP used. The shape of a NP has not been proven to be a crucial factor influencing its anti-microbial activity. Some authors have shown that AgNPs with a truncated triangular shape, or similar geometry such as an hexagonal and octahedral shape, are more effective against bacteria, while other authors have reported that the shape of an AgNP does not have any influence on its activity [36–38]. Recent publications showed that NPs can prolong the ocular retention of some topical drugs, thus enabling treatment of eye diseases using reduced drug dosages [39, 40]. It was confirmed that NPs coated onto the surface of contact lenses caused a significant reduction in the microbial colonization of the surface [41]. After 6 h of incubation, contact lenses impregnated with AgNPs did not exhibit desirable anti-bacterial activity against *Staphylococcus aureus*, although excellent anti-bacterial effects against *Pseudomonas aeruginosa* were demonstrated [42]. Silver-impregnated lens cases showed less microbial contamination compared to control cases. Most microorganisms isolated from silver-impregnated cases were members of the normal skin flora [43].

Only a few studies have examined the activity of NPs against *Acanthamoeba* spp. Cobalt NPs have been studied for their anti-amoebic potential, and hexagonal microflakes showed the most promising anti-*Acanthamoeba* effects compared to nanoflakes and granular cobalt NPs. Apart from their concentration and size, the composition and morphology of the tested noncompounds also determined their anti-amoebic activity [44, 45]. AgNPs are well absorbed by *Acanthamoeba* trophozoites and integrated into the cell matrix. NPs decrease the viability of trophozoites and alter their metabolic activity in a dose-dependent manner [46]. In our previous studies we confirmed that AgNPs conjugated with contact lens solutions showed dose-dependent enhanced anti-amoebic activity [47]. Recently published studies confirmed the enhanced anti-microbial effects of AgNPs and gold NPs (AuNPs) conjugated with commonly used drugs like chlorhexidine, fluconazole or amphotericin B, as well as with some disinfectants [27, 48]. Guanabenz, a drug that crosses the blood-brain barrier and has already been approved for the treatment of hypertension, showed significant anti-amoebic activity against both *A. castellanii* and *Naegleria fowleri* when conjugated with AuNPs and AgNPs. A significant reduction in host cytopathogenicity, especially for silver nanoconjugates, was revealed, and was associated with negligible cytotoxicity against human cells [49].

Environmentally friendly and cost-effective bio-nanotechnology techniques are being developed to produce anti-microbial active conjugates as potential candidates for the eradication of infections and reduction of microbial contamination of medical devices including contact lenses. Products created by the integration and conjugation of bioactive agents with nanomaterials have been tested mainly for their anti-bacterial activities. AgNPs, AuNPs and platinum NPs produced by green synthesis showed enhanced anti-bacterial activity after combination with different classes of antibiotics [50]. The biosynthesis of AgNPs with an extract of *Salvia spinosa* resulted in increased bactericidal activity against Gram-positive and Gram-negative bacteria [51]. Novel conjugates using biogenic AgNPs from *Convolvulus arvensi* extract and chitosan showed anti-microbial, anti-biofilm, and anti-cancer potentialities [52]. An extract of *Oscillatoria limnetica* conjugated with AgNPs exhibited strong anti-bacterial activity against multidrug-resistant bacteria as well as cytotoxic effects against both a human breast cancer cell line and a human colon cancer cell line [53]. Synthesis of silver chloride NPs, using walnut green husk extract as well as AgNPs with *Peganum harmala* L. leaf extract resulted in significant inhibitory effects against clinical isolates of *Escherichia coli* and *S. aureus* [54, 55]. Bio-nanotechnology has not been extensively studied on protozoan species. There are just a few published studies focusing on the influence of NPs conjugated with plant extracts on amoebae. *Jatropha curcas*, *Jatropha gossypifolia* and *Euphorbia milii* extracts combined with NPs exhibited a significant reduction of *Acanthamoeba* trophozoites, with a low cytotoxic effect on human cells [25]. In our previous studies we confirmed that AgTANPs showed higher anti-amoebic activity and less cytotoxicity to human cells in comparison with pure AgNPs [24]. In the present study we revealed that AgTANPs conjugated with contact lens solutions exhibited even better anti-amoebic activity in relation to cytotoxicity than in our previous studies where we tested pure AgNP conjugates [47]. We conclude that differences in the anti-amoebic activity of the tested conjugates may be mainly driven by the anti-amoebic activity of the pure contact lens solutions. NPs at the tested concentration seem to enhance the existing anti-amoebic potential of the selected contact lens solutions.

## Conclusions

In this study, we showed dose-dependent enhancement of the anti-amoebic effect of AgTANPs conjugated with SCA and ReNu solutions against an *Acanthamoeba* T4 strain. These promising results were obtained within the minimal disinfection time recommended by the manufacturers (6 h) and without increased toxicity to human cells. In summary, conjugation of the selected contact lens solutions with AgTANPs might be a promising approach for the prevention of AK infections in contact lens users. However, further studies should be conducted to elucidate the stability of the conjugates and their activity against *Acanthamoeba* spp*.* cysts.

## Data Availability

The datasets used and/or analysed during the current study are available from the corresponding author on reasonable request.

## Abbreviations

AK: *Acanthamoeba* keratitis
NPs: Nanoparticles
AgTANPs: Tannic acid-modified silver nanoparticles
HR-STEM: High-resolution scanning transmission electron microscopy
SCA: Solo Care Aqua
O-F: Opti-Free
ReNu: ReNu MultiPlus
AgNPs: Silver nanoparticles
AuNPs: Gold nanoparticles
